# Editorial for the Special Issue on Organs-on-Chips

**DOI:** 10.3390/mi11040369

**Published:** 2020-04-01

**Authors:** Yu-suke Torisawa, Yi-Chung Tung

**Affiliations:** 1 Hakubi Center for Advanced Research, Kyoto University, Kyoto 615-8540, Japan; 2 Department of Micro Engineering, Kyoto University, Kyoto 615-8540, Japan; 3 Research Center for Applied Sciences, Academia Sinica, Taipei 11529, Taiwan; 4 College of Engineering, Chang Gung University, Taoyuan 33302, Taiwan

Recent advances in microsystems technology and cell culture techniques have led to the development of organ-on-chip microdevices to model functional units of organs. By recapitulating natural tissue architecture and microenvironmental chemical and mechanical cues, organs-on-chips reconstitute tissue-level functionalities in vitro, which is infeasible with conventional culture methods. Organs-on-chips are typically microfluidic culture devices made of optically transparent materials that permit high-resolution imaging and real-time monitoring of cellar responses. These microdevices allow to mimic the biomechanical forces observed inside the body, playing important roles in controlling cell fates and functions. Recently, stem cell technologies including induced pluripotent stem (iPS) cells have been leveraged to develop organs-on-chips, which enable various types of models of organs and diseases not possible with primary cells and cell lines. Organs-on-chips replicate tissue-level responses with human cells, enabling more accurate prediction of human responses to drugs and diseases. Since the cost of drug discovery is constantly increasing due to the limited predictability of conventional monolayer culture methods and animal models, this technology has great potential to promote drug discovery and development as well as to model human physiology and disease.

This Special Issue is themed to provide insight and advancements in organ-on-chip microdevices. There are fifteen papers including three review papers, covering a novel material to fabricate microfluidic organs-on-chips [1], methods to deliver mechanical stimuli [2,3], methods to measure mechanical forces [4,5], methods to evaluate cellular functions in 3D cultures [6,7,8], and specific organ models; lung chips [3,9], liver chips [10,11], blood vessel chips [12,13,14,15] including models of the outer blood-retina barrier [14] and ischemia-reperfusion injury [15].

Inside the body, cells are exposed to biomechanical forces, including fluidic shear stress and mechanical strain, which regulate cell function and contribute to disease. Kaarj et al. reviewed methods to produce mechanical stimuli focusing on the technical details of devices [2]. This paper shows organ-on-chip systems that incorporate various types of mechanical stimuli and their potential applications to develop physiologically relevant models and to study mechanobiology. Lin et al. developed a simple yet powerful microfluidic device that can generate hydrostatic pressure and cyclic strain to mimic the lung physiological microenvironment [3]. This device paves the way to better understand the cellular behaviors under various lung physiological conditions for future translational studies. It is also important to quantitatively measure mechanical forces generated by cells and tissues. A strain gauge has been developed to measure mechanical strain in situ [4]. This strain gauge can be used to measure mechanical strain on a membrane where cells are cultured. This method enables the integration of the strain gauges in monolithically fabricated organ-on-chip devices. Another approach using a micro vacuum chuck has been developed to measure biomechanical properties of a three-dimensional (3D) tissue [5]. This tool can position a cardiac tissue by vacuum pressure, which enables the measurement of beating force of a cardiac tissue assembled from human iPSC-derived cardiomyocytes. These methods to measure mechanical forces can be useful for heart-on-chip applications.

By combining 3D cell culture and microdevice technologies, 3D culture devices have been developed to replicate in vivo-like microenvironment as well as to develop high-throughput systems. Bastiaens et al. developed a 3D neuronal culture device by combining nanogrooved substrates with a 3D hydrogel culture [6]. This method permits the formation of an aligned 3D neural cell network. The use of nanogrooves enhances the structural complexity of 3D neuronal cell cultures, providing a way to develop a brain-on-a-chip model. Chen et al. developed an imaging method for 3D cultures [7]. By using lattice light-sheet microscopy, individual neuronal cells in a 3D hippocampal neuron can be monitored. This method makes quantification of voltage responses and calcium dynamics in individual neurons in 3D culture feasible. Choi et al. developed a chip to form an array of 3D cell spheroids for drug testing [8]. A 12-by-36 array of alginate hydrogels containing cancer spheroids was formed using micropillars and microwells. This system can test seventy drugs at six replicates on a chip providing a useful platform for drug screening.

Various types of organs-on-chips have been reported in this issue. Frost et al. reported a microfluidic lung-on-a-chip by recreating the epithelial-endothelial interface of the lung to evaluate drug permeability [9]. This microfluidic device allows to evaluate the effect of fluid shear stress on tissue permeability. Deng et al. reviewed strategies to build liver-on-chip models [10]. Liver chips consisting of human cells could potentially correlate clinical testing. These chips enable to predict hepatotoxicity and metabolism of drugs in humans and can be connected to other organ chips to recapitulate physiological interactions between multiple organs. A biomimetic method has also been developed to mimic drug metabolism in the liver [11]. Catalysts immobilized onto magnetic nanoparticles could efficiently produce drug metabolites in very small volumes.

Blood vessel chips have been developed to study angiogenesis and to develop disease models. Wang et al. reviewed current strategies to engineer microvessels on-chip focusing on the generation of 3D microvascular networks [12]. Akbari et al. reported the role of the flow conditions that occur due to vessel bifurcations on endothelial sprouting using a microfluidic 3D culture device [13]. This model revealed the importance of local flow dynamics due to branched vessel geometry in determining the location of sprouting angiogenesis. A microfluidic co-culture model has been developed to recapitulate the outer blood-retina barrier [14]. The device consists of an upper microchannel and multiple lower microchannels to form co-culture with 3D blood vessels. By integrating platinum electrodes into the device, this system allows to measure trans-epithelial electrical resistance (TEER) in real time, enabling to assess the epithelial barrier integrity on-chip. Nemcovsky et al. developed a novel microfluidic system to model ischemia-reperfusion injury [15]. This system consists of a vascular compartment lined with human endothelial cells that can be obstructed with a human blood clot and then re-perfused by thrombolytic treatment. Restoration of blood supply is essential to salvage ischemic tissue; however, reperfusion paradoxically causes further damage, even in remote tissues. The microfluidic mode of ischemia-reperfusion injury permits to recapitulate key features following restoration of flow upon removal of vascular embolic occlusion and thus this system can potentially serve as a powerful platform to study new therapeutic approaches for treatment of ischemia-reperfusion injury.

These organs-on-chips are mostly made of polydimethylsiloxane (PDMS) because it is easy to use, biocompatible, highly gas permeable, optically clear, and flexible. Although PDMS is very useful, one serious drawback is that small hydrophobic molecules are strongly absorbed into PDMS. This limitation is critical for drug testing because PDMS soaks up small hydrophobic drugs. Sano et al. reported a novel method to fabricate microfluidic devices using a fluoroelastomer which is resistant to absorption of small hydrophobic drugs comparable with standard culture plates [1]. This method could be a useful platform to construct organs-on-chips for drug discovery and development. Since organs-on-chips have now attracted great attention from the pharmaceutical industry, it is very important to identify suitable materials to develop commercialization-ready organs-on-chips.

We thank all the authors who submitted their papers to this Special Issue. We would also like to acknowledge all the reviewers whose careful and timely reviews ensured the quality of this Special Issue.
